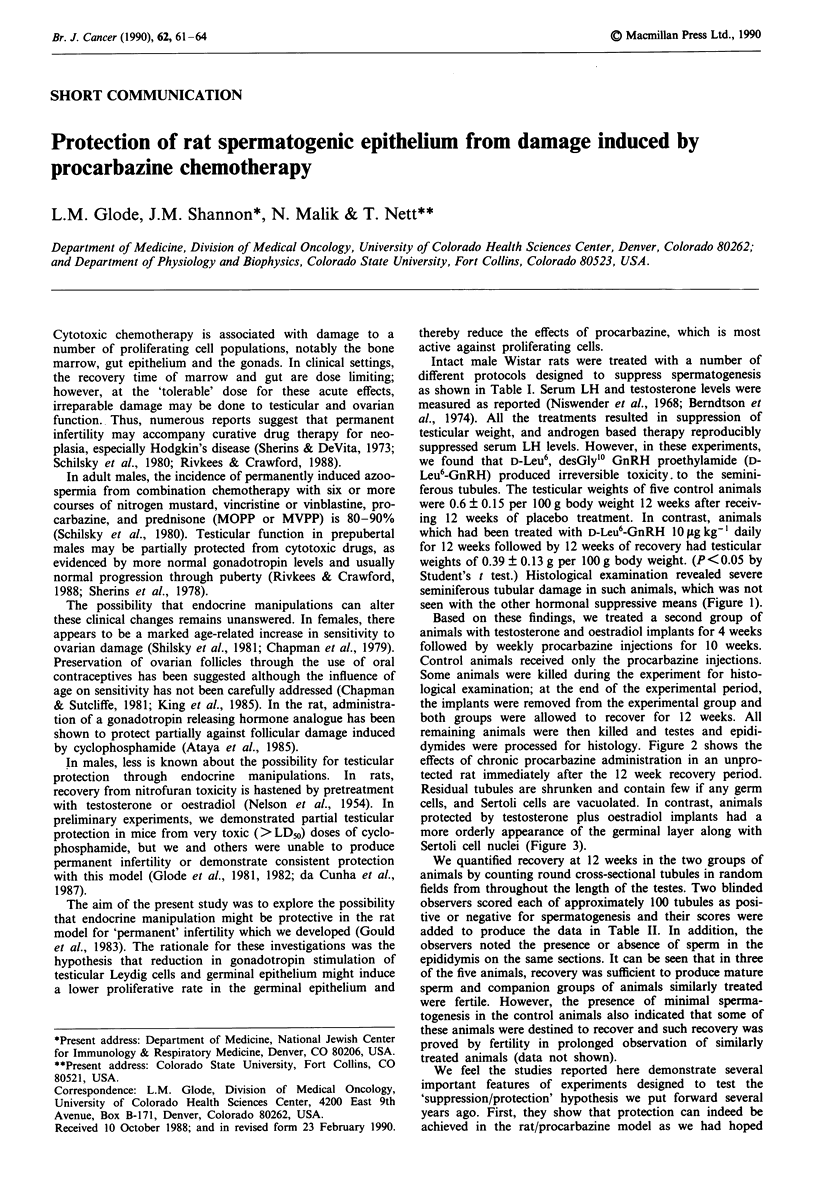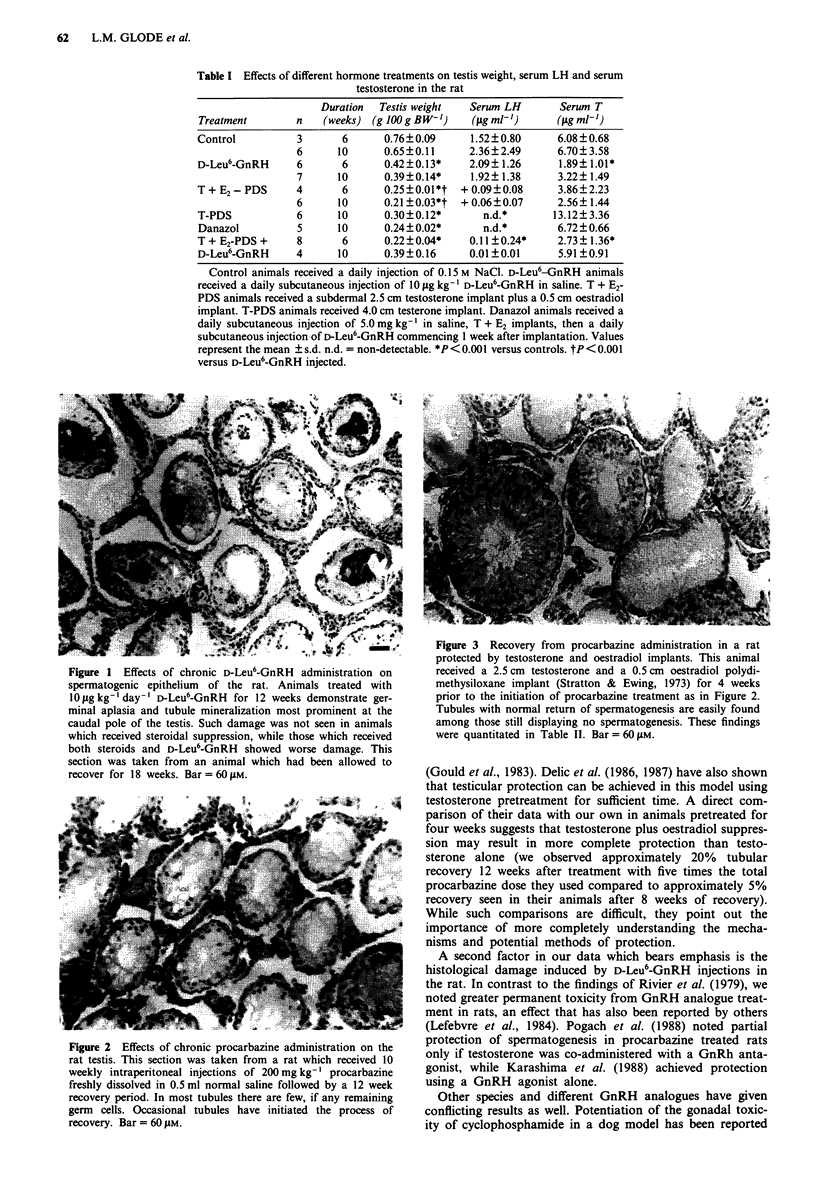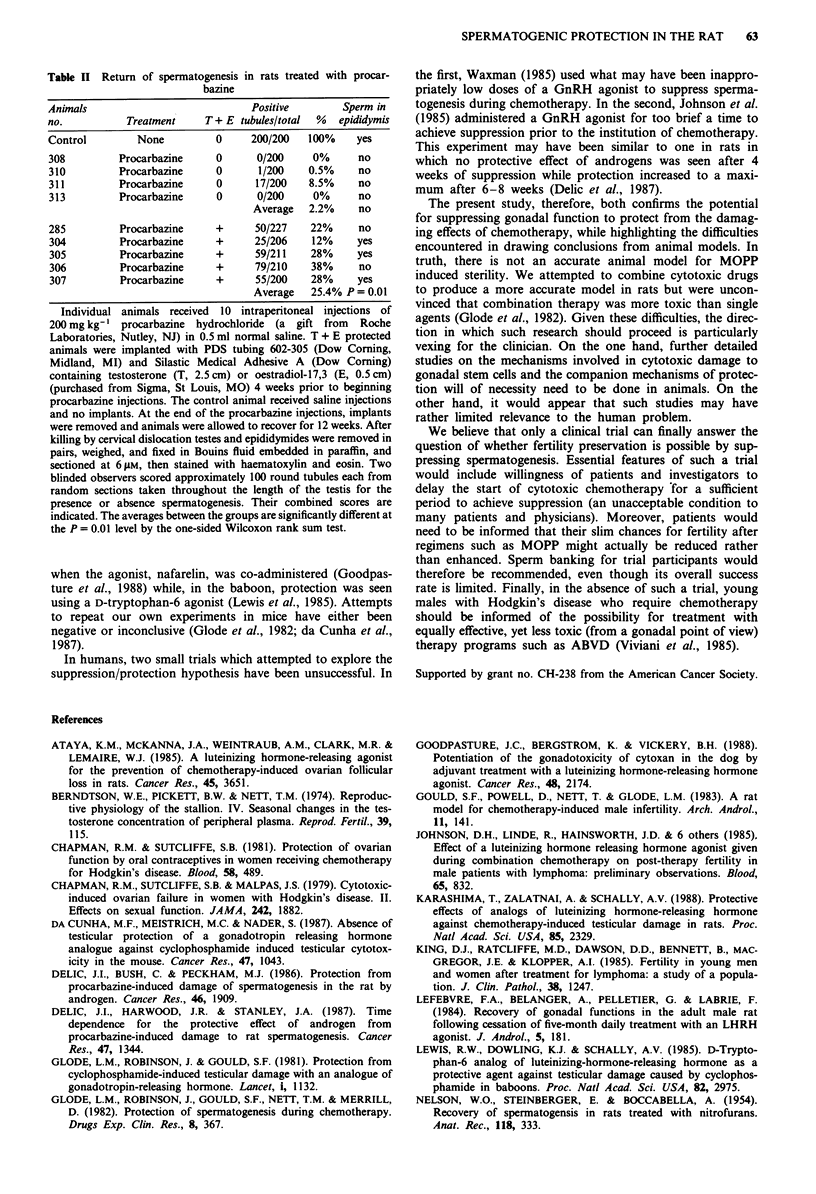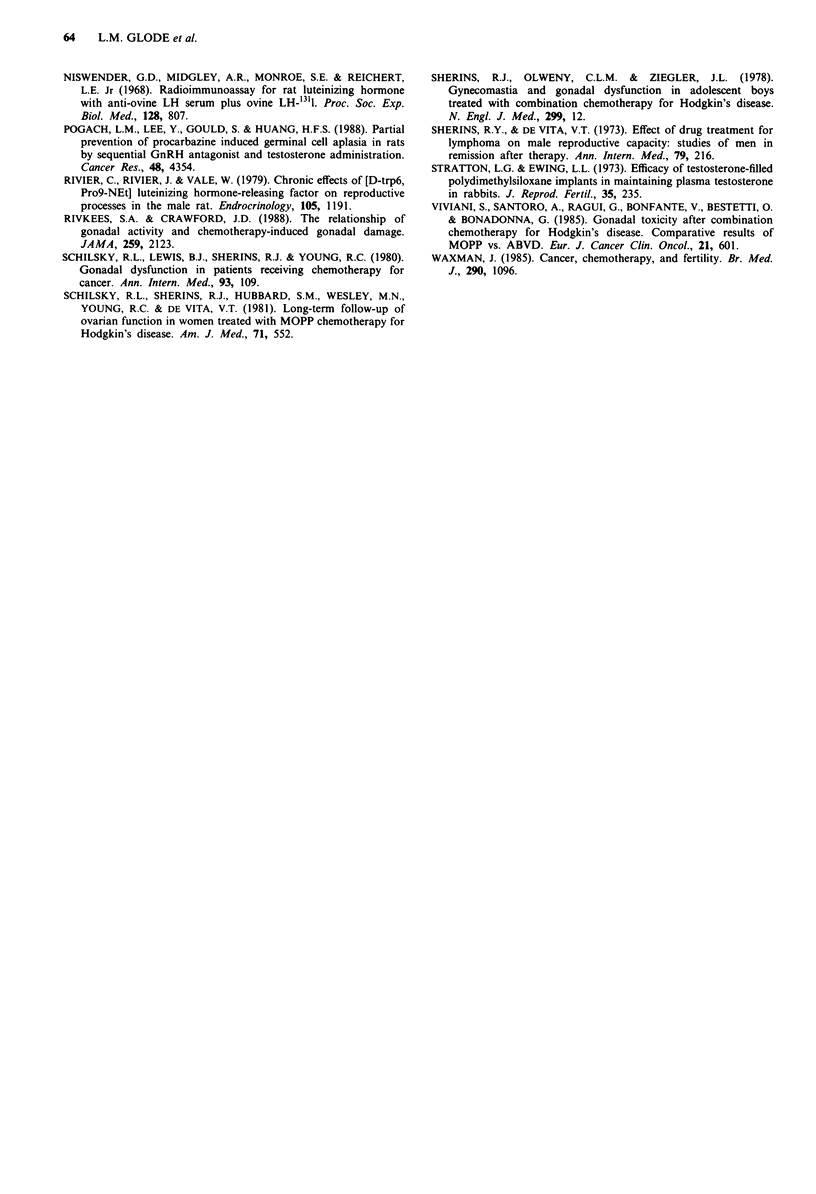# Protection of rat spermatogenic epithelium from damage induced by procarbazine chemotherapy.

**DOI:** 10.1038/bjc.1990.229

**Published:** 1990-07

**Authors:** L. M. Glode, J. M. Shannon, N. Malik, T. Nett

**Affiliations:** Department of Medicine, University of Colorado Health Sciences Center, Denver 80262.

## Abstract

**Images:**


Br. J. Cancer (1990), 62, 61-64 © Macmillan Press Ltd., 1990

SHORT COMMUNICATION

### Protection of rat spermatogenic epithelium from damage induced by procarbazine chemotherapy

L.M. Glode, J.M. Shannon*, N. Malik & T. Nett**

*Department of Medicine, Division of Medical Oncology, University of Colorado Health Sciences Center, Denver, Colorado 80262; and Department of Physiology and Biophysics, Colorado State University, Fort Collins, Colorado 80523, USA.*

Cytotoxic chemotherapy is associated with damage to a number of proliferating cell populations, notably the bone marrow, gut epithelium and the gonads. In clinical settings, the recovery time of marrow and gut are dose limiting; however, at the 'tolerable' dose for these acute effects, irreparable damage may be done to testicular and ovarian function. Thus, numerous reports suggest that permanent infertility may accompany curative drug therapy for neoplasia, especially Hodgkin's disease (Sherins & DeVita, 1973; Schilsky *et al.*, 1980; Rivkees & Crawford, 1988).

In adult males, the incidence of permanently induced azoospermia from combination chemotherapy with six or more courses of nitrogen mustard, vincristine or vinblastine, procarbazine, and prednisone (MOPP or MVPP) is 80-90% (Schilsky *et al.*, 1980). Testicular function in prepubertal males may be partially protected from cytotoxic drugs, as evidenced by more normal gonadotropin levels and usually normal progression through puberty (Rivkees & Crawford, 1988; Sherins *et al.*, 1978).

The possibility that endocrine manipulations can alter these clinical changes remains unanswered. In females, there appears to be a marked age-related increase in sensitivity to ovarian damage (Shilsky *et al.*, 1981; Chapman *et al.*, 1979). Preservation of ovarian follicles through the use of oral contraceptives has been suggested although the influence of age on sensitivity has not been carefully addressed (Chapman & Sutcliffe, 1981; King *et al.*, 1985). In the rat, administration of a gonadotropin releasing hormone analogue has been shown to protect partially against follicular damage induced by cyclophosphamide (Ataya *et al.*, 1985).

In males, less is known about the possibility for testicular protection through endocrine manipulations. In rats, recovery from nitrofuran toxicity is hastened by pretreatment with testosterone or oestradiol (Nelson *et al.*, 1954). In preliminary experiments, we demonstrated partial testicular protection in mice from very toxic ($>LD_{50}$) doses of cyclophosphamide, but we and others were unable to produce permanent infertility or demonstrate consistent protection with this model (Glode *et al.*, 1981, 1982; da Cunha *et al.*, 1987).

The aim of the present study was to explore the possibility that endocrine manipulation might be protective in the rat model for 'permanent' infertility which we developed (Gould *et al.*, 1983). The rationale for these investigations was the hypothesis that reduction in gonadotropin stimulation of testicular Leydig cells and germinal epithelium might induce a lower proliferative rate in the germinal epithelium and thereby reduce the effects of procarbazine, which is most active against proliferating cells.

Intact male Wistar rats were treated with a number of different protocols designed to suppress spermatogenesis as shown in Table I. Serum LH and testosterone levels were measured as reported (Niswender *et al.*, 1968; Berndtson *et al.*, 1974). All the treatments resulted in suppression of testicular weight, and androgen based therapy reproducibly suppressed serum LH levels. However, in these experiments, we found that D-Leu$^6$, desGly$^{10}$ GnRH proethylamide (D-Leu$^6$-GnRH) produced irreversible toxicity to the seminiferous tubules. The testicular weights of five control animals were $0.6 \pm 0.15$ per 100 g body weight 12 weeks after receiving 12 weeks of placebo treatment. In contrast, animals which had been treated with D-Leu$^6$-GnRH 10 $\mu g\,kg^{-1}$ daily for 12 weeks followed by 12 weeks of recovery had testicular weights of $0.39 \pm 0.13$ g per 100 g body weight. ($P<0.05$ by Student's *t* test.) Histological examination revealed severe seminiferous tubular damage in such animals, which was not seen with the other hormonal suppressive means (Figure 1).

Based on these findings, we treated a second group of animals with testosterone and oestradiol implants for 4 weeks followed by weekly procarbazine injections for 10 weeks. Control animals received only the procarbazine injections. Some animals were killed during the experiment for histological examination; at the end of the experimental period, the implants were removed from the experimental group and both groups were allowed to recover for 12 weeks. All remaining animals were then killed and testes and epididymides were processed for histology. Figure 2 shows the effects of chronic procarbazine administration in an unprotected rat immediately after the 12 week recovery period. Residual tubules are shrunken and contain few if any germ cells, and Sertoli cells are vacuolated. In contrast, animals protected by testosterone plus oestradiol implants had a more orderly appearance of the germinal layer along with Sertoli cell nuclei (Figure 3).

We quantified recovery at 12 weeks in the two groups of animals by counting round cross-sectional tubules in random fields from throughout the length of the testes. Two blinded observers scored each of approximately 100 tubules as positive or negative for spermatogenesis and their scores were added to produce the data in Table II. In addition, the observers noted the presence or absence of sperm in the epididymis on the same sections. It can be seen that in three of the five animals, recovery was sufficient to produce mature sperm and companion groups of animals similarly treated were fertile. However, the presence of minimal spermatogenesis in the control animals also indicated that some of these animals were destined to recover and such recovery was proved by fertility in prolonged observation of similarly treated animals (data not shown).

We feel the studies reported here demonstrate several important features of experiments designed to test the 'suppression/protection' hypothesis we put forward several years ago. First, they show that protection can indeed be achieved in the rat/procarbazine model as we had hoped

*Present address: Department of Medicine, National Jewish Center for Immunology & Respiratory Medicine, Denver, CO 80206, USA.
**Present address: Colorado State University, Fort Collins, CO 80521, USA.
Correspondence: L.M. Glode, Division of Medical Oncology, University of Colorado Health Sciences Center, 4200 East 9th Avenue, Box B-171, Denver, Colorado 80262, USA.
Received 10 October 1988; and in revised form 23 February 1990.

**Table I** Effects of different hormone treatments on testis weight, serum LH and serum testosterone in the rat

| *Treatment* | *n* | *Duration (weeks)* | *Testis weight (g 100 g BW$^{-1}$)* | *Serum LH (μg ml$^{-1}$)* | *Serum T (μg ml$^{-1}$)* |
|---|---|---|---|---|---|
| Control | 3 | 6 | 0.76±0.09 | 1.52±0.80 | 6.08±0.68 |
| | 6 | 10 | 0.65±0.11 | 2.36±2.49 | 6.70±3.58 |
| D-Leu$^{6}$-GnRH | 6 | 6 | 0.42±0.13* | 2.09±1.26 | 1.89±1.01* |
| | 7 | 10 | 0.39±0.14* | 1.92±1.38 | 3.22±1.49 |
| T + $E_2$ – PDS | 4 | 6 | 0.25±0.01*† | +0.09±0.08 | 3.86±2.23 |
| | 6 | 10 | 0.21±0.03*† | +0.06±0.07 | 2.56±1.44 |
| T-PDS | 6 | 10 | 0.30±0.12* | n.d.* | 13.12±3.36 |
| Danazol | 5 | 10 | 0.24±0.02* | n.d.* | 6.72±0.66 |
| T + $E_2$-PDS + | 8 | 6 | 0.22±0.04* | 0.11±0.24* | 2.73±1.36* |
| D-Leu$^{6}$-GnRH | 4 | 10 | 0.39±0.16 | 0.01±0.01 | 5.91±0.91 |

Control animals received a daily injection of 0.15 M NaCl. D-Leu$^{6}$–GnRH animals received a daily subcutaneous injection of 10 μg kg$^{-1}$ D-Leu$^{6}$-GnRH in saline. T + $E_2$-PDS animals received a subdermal 2.5 cm testosterone implant plus a 0.5 cm oestradiol implant. T-PDS animals received 4.0 cm testerone implant. Danazol animals received a daily subcutaneous injection of 5.0 mg kg$^{-1}$ in saline, T + $E_2$ implants, then a daily subcutaneous injection of D-Leu$^{6}$-GnRH commencing 1 week after implantation. Values represent the mean ±s.d. n.d. = non-detectable. *$P<0.001$ versus controls. †$P<0.001$ versus D-Leu$^{6}$-GnRH injected.

**Figure 1** Effects of chronic D-Leu$^{6}$-GnRH administration on spermatogenic epithelium of the rat. Animals treated with 10 μg kg$^{-1}$ day$^{-1}$ D-Leu$^{6}$-GnRH for 12 weeks demonstrate germinal aplasia and tubule mineralization most prominent at the caudal pole of the testis. Such damage was not seen in animals which received steroidal suppression, while those which received both steroids and D-Leu$^{6}$-GnRH showed worse damage. This section was taken from an animal which had been allowed to recover for 18 weeks. Bar = 60 μM.

**Figure 2** Effects of chronic procarbazine administration on the rat testis. This section was taken from a rat which received 10 weekly intraperitoneal injections of 200 mg kg$^{-1}$ procarbazine freshly dissolved in 0.5 ml normal saline followed by a 12 week recovery period. In most tubules there are few, if any remaining germ cells. Occasional tubules have initiated the process of recovery. Bar = 60 μM.

**Figure 3** Recovery from procarbazine administration in a rat protected by testosterone and oestradiol implants. This animal received a 2.5 cm testosterone and a 0.5 cm oestradiol polydimethysiloxane implant (Stratton & Ewing, 1973) for 4 weeks prior to the initiation of procarbazine treatment as in Figure 2. Tubules with normal return of spermatogenesis are easily found among those still displaying no spermatogenesis. These findings were quantitated in Table II. Bar = 60 μM.

(Gould *et al.*, 1983). Delic *et al.* (1986, 1987) have also shown that testicular protection can be achieved in this model using testosterone pretreatment for sufficient time. A direct comparison of their data with our own in animals pretreated for four weeks suggests that testosterone plus oestradiol suppression may result in more complete protection than testosterone alone (we observed approximately 20% tubular recovery 12 weeks after treatment with five times the total procarbazine dose they used compared to approximately 5% recovery seen in their animals after 8 weeks of recovery). While such comparisons are difficult, they point out the importance of more completely understanding the mechanisms and potential methods of protection.

A second factor in our data which bears emphasis is the histological damage induced by D-Leu$^{6}$-GnRH injections in the rat. In contrast to the findings of Rivier *et al.* (1979), we noted greater permanent toxicity from GnRH analogue treatment in rats, an effect that has also been reported by others (Lefebvre *et al.*, 1984). Pogach *et al.* (1988) noted partial protection of spermatogenesis in procarbazine treated rats only if testosterone was co-administered with a GnRh antagonist, while Karashima *et al.* (1988) achieved protection using a GnRH agonist alone.

Other species and different GnRH analogues have given conflicting results as well. Potentiation of the gonadal toxicity of cyclophosphamide in a dog model has been reported

**Table II** Return of spermatogenesis in rats treated with procarbazine

| *Animals no.* | *Treatment* | *T + E* | *Positive tubules/total* | *%* | *Sperm in epididymis* |
|---|---|---|---|---|---|
| Control | None | 0 | 200/200 | 100% | yes |
| 308 | Procarbazine | 0 | 0/200 | 0% | no |
| 310 | Procarbazine | 0 | 1/200 | 0.5% | no |
| 311 | Procarbazine | 0 | 17/200 | 8.5% | no |
| 313 | Procarbazine | 0 | 0/200 | 0% | no |
| | | | Average | 2.2% | no |
| 285 | Procarbazine | + | 50/227 | 22% | no |
| 304 | Procarbazine | + | 25/206 | 12% | yes |
| 305 | Procarbazine | + | 59/211 | 28% | yes |
| 306 | Procarbazine | + | 79/210 | 38% | no |
| 307 | Procarbazine | + | 55/200 | 28% | yes |
| | | | Average | 25.4% | $P = 0.01$ |

Individual animals received 10 intraperitoneal injections of 200 mg $kg^{-1}$ procarbazine hydrochloride (a gift from Roche Laboratories, Nutley, NJ) in 0.5 ml normal saline. T + E protected animals were implanted with PDS tubing 602-305 (Dow Corning, Midland, MI) and Silastic Medical Adhesive A (Dow Corning) containing testosterone (T, 2.5 cm) or oestradiol-17,3 (E, 0.5 cm) (purchased from Sigma, St Louis, MO) 4 weeks prior to beginning procarbazine injections. The control animal received saline injections and no implants. At the end of the procarbazine injections, implants were removed and animals were allowed to recover for 12 weeks. After killing by cervical dislocation testes and epididymides were removed in pairs, weighed, and fixed in Bouins fluid embedded in paraffin, and sectioned at 6 μM, then stained with haematoxylin and eosin. Two blinded observers scored approximately 100 round tubules each from random sections taken throughout the length of the testis for the presence or absence spermatogenesis. Their combined scores are indicated. The averages between the groups are significantly different at the $P = 0.01$ level by the one-sided Wilcoxon rank sum test.

when the agonist, nafarelin, was co-administered (Goodpasture *et al.*, 1988) while, in the baboon, protection was seen using a D-tryptophan-6 agonist (Lewis *et al.*, 1985). Attempts to repeat our own experiments in mice have either been negative or inconclusive (Glode *et al.*, 1982; da Cunha *et al.*, 1987).

In humans, two small trials which attempted to explore the suppression/protection hypothesis have been unsuccessful. In the first, Waxman (1985) used what may have been inappropriately low doses of a GnRH agonist to suppress spermatogenesis during chemotherapy. In the second, Johnson *et al.* (1985) administered a GnRH agonist for too brief a time to achieve suppression prior to the institution of chemotherapy. This experiment may have been similar to one in rats in which no protective effect of androgens was seen after 4 weeks of suppression while protection increased to a maximum after 6–8 weeks (Delic *et al.*, 1987).

The present study, therefore, both confirms the potential for suppressing gonadal function to protect from the damaging effects of chemotherapy, while highlighting the difficulties encountered in drawing conclusions from animal models. In truth, there is not an accurate animal model for MOPP induced sterility. We attempted to combine cytotoxic drugs to produce a more accurate model in rats but were unconvinced that combination therapy was more toxic than single agents (Glode *et al.*, 1982). Given these difficulties, the direction in which such research should proceed is particularly vexing for the clinician. On the one hand, further detailed studies on the mechanisms involved in cytotoxic damage to gonadal stem cells and the companion mechanisms of protection will of necessity need to be done in animals. On the other hand, it would appear that such studies may have rather limited relevance to the human problem.

We believe that only a clinical trial can finally answer the question of whether fertility preservation is possible by suppressing spermatogenesis. Essential features of such a trial would include willingness of patients and investigators to delay the start of cytotoxic chemotherapy for a sufficient period to achieve suppression (an unacceptable condition to many patients and physicians). Moreover, patients would need to be informed that their slim chances for fertility after regimens such as MOPP might actually be reduced rather than enhanced. Sperm banking for trial participants would therefore be recommended, even though its overall success rate is limited. Finally, in the absence of such a trial, young males with Hodgkin's disease who require chemotherapy should be informed of the possibility for treatment with equally effective, yet less toxic (from a gonadal point of view) therapy programs such as ABVD (Viviani *et al.*, 1985).

Supported by grant no. CH-238 from the American Cancer Society.

### References

ATAYA, K.M., MCKANNA, J.A., WEINTRAUB, A.M., CLARK, M.R. & LEMAIRE, W.J. (1985). A luteinizing hormone-releasing agonist for the prevention of chemotherapy-induced ovarian follicular loss in rats. *Cancer Res.*, **45**, 3651.

BERNDTSON, W.E., PICKETT, B.W. & NETT, T.M. (1974). Reproductive physiology of the stallion. IV. Seasonal changes in the testosterone concentration of peripheral plasma. *Reprod. Fertil.*, **39**, 115.

CHAPMAN, R.M. & SUTCLIFFE, S.B. (1981). Protection of ovarian function by oral contraceptives in women receiving chemotherapy for Hodgkin's disease. *Blood*, **58**, 489.

CHAPMAN, R.M., SUTCLIFFE, S.B. & MALPAS, J.S. (1979). Cytotoxic-induced ovarian failure in women with Hodgkin's disease. II. Effects on sexual function. *JAMA*, **242**, 1882.

DA CUNHA, M.F., MEISTRICH, M.C. & NADER, S. (1987). Absence of testicular protection of a gonadotropin releasing hormone analogue against cyclophosphamide induced testicular cytotoxicity in the mouse. *Cancer Res.*, **47**, 1043.

DELIC, J.I., BUSH, C. & PECKHAM, M.J. (1986). Protection from procarbazine-induced damage of spermatogenesis in the rat by androgen. *Cancer Res.*, **46**, 1909.

DELIC, J.I., HARWOOD, J.R. & STANLEY, J.A. (1987). Time dependence for the protective effect of androgen from procarbazine-induced damage to rat spermatogenesis. *Cancer Res.*, **47**, 1344.

GLODE, L.M., ROBINSON, J. & GOULD, S.F. (1981). Protection from cyclophosphamide-induced testicular damage with an analogue of gonadotropin-releasing hormone. *Lancet*, **i**, 1132.

GLODE, L.M., ROBINSON, J., GOULD, S.F., NETT, T.M. & MERRILL, D. (1982). Protection of spermatogenesis during chemotherapy. *Drugs Exp. Clin. Res.*, **8**, 367.

GOODPASTURE, J.C., BERGSTROM, K. & VICKERY, B.H. (1988). Potentiation of the gonadotoxicity of cytoxan in the dog by adjuvant treatment with a luteinizing hormone-releasing hormone agonist. *Cancer Res.*, **48**, 2174.

GOULD, S.F., POWELL, D., NETT, T. & GLODE, L.M. (1983). A rat model for chemotherapy-induced male infertility. *Arch. Androl.*, **11**, 141.

JOHNSON, D.H., LINDE, R., HAINSWORTH, J.D. & 6 others (1985). Effect of a luteinizing hormone releasing hormone agonist given during combination chemotherapy on post-therapy fertility in male patients with lymphoma: preliminary observations. *Blood*, **65**, 832.

KARASHIMA, T., ZALATNAI, A. & SCHALLY, A.V. (1988). Protective effects of analogs of luteinizing hormone-releasing hormone against chemotherapy-induced testicular damage in rats. *Proc. Natl Acad. Sci. USA*, **85**, 2329.

KING, D.J., RATCLIFFE, M.D., DAWSON, D.D., BENNETT, B., MACGREGOR, J.E. & KLOPPER, A.I. (1985). Fertility in young men and women after treatment for lymphoma: a study of a population. *J. Clin. Pathol.*, **38**, 1247.

LEFEBVRE, F.A., BELANGER, A., PELLETIER, G. & LABRIE, F. (1984). Recovery of gonadal functions in the adult male rat following cessation of five-month daily treatment with an LHRH agonist. *J. Androl.*, **5**, 181.

LEWIS, R.W., DOWLING, K.J. & SCHALLY, A.V. (1985). D-Tryptophan-6 analog of luteinizing-hormone-releasing hormone as a protective agent against testicular damage caused by cyclophosphamide in baboons. *Proc. Natl Acad. Sci. USA*, **82**, 2975.

NELSON, W.O., STEINBERGER, E. & BOCCABELLA, A. (1954). Recovery of spermatogensis in rats treated with nitrofurans. *Anat. Rec.*, **118**, 333.

NISWENDER, G.D., MIDGLEY, A.R., MONROE, S.E. & REICHERT, L.E. Jr (1968). Radioimmunoassay for rat luteinizing hormone with anti-ovine LH serum plus ovine LH-$^{131}$I. *Proc. Soc. Exp. Biol. Med.*, **128,** 807.

POGACH, L.M., LEE, Y., GOULD, S. & HUANG, H.F.S. (1988). Partial prevention of procarbazine induced germinal cell aplasia in rats by sequential GnRH antagonist and testosterone administration. *Cancer Res.*, **48,** 4354.

RIVIER, C., RIVIER, J. & VALE, W. (1979). Chronic effects of [D-trp6, Pro9-NEt] luteinizing hormone-releasing factor on reproductive processes in the male rat. *Endrocrinology*, **105,** 1191.

RIVKEES, S.A. & CRAWFORD, J.D. (1988). The relationship of gonadal activity and chemotherapy-induced gonadal damage. *JAMA*, **259,** 2123.

SCHILSKY, R.L., LEWIS, B.J., SHERINS, R.J. & YOUNG, R.C. (1980). Gonadal dysfunction in patients receiving chemotherapy for cancer. *Ann. Intern. Med.*, **93,** 109.

SCHILSKY, R.L., SHERINS, R.J., HUBBARD, S.M., WESLEY, M.N., YOUNG, R.C. & DE VITA, V.T. (1981). Long-term follow-up of ovarian function in women treated with MOPP chemotherapy for Hodgkin's disease. *Am. J. Med.*, **71,** 552.

SHERINS, R.J., OLWENY, C.L.M. & ZIEGLER, J.L. (1978). Gynecomastia and gonadal dysfunction in adolescent boys treated with combination chemotherapy for Hodgkin's disease. *N. Engl. J. Med.*, **299,** 12.

SHERINS, R.Y., & DE VITA, V.T. (1973). Effect of drug treatment for lymphoma on male reproductive capacity: studies of men in remission after therapy. *Ann. Intern. Med.*, **79,** 216.

STRATTON, L.G. & EWING, L.L. (1973). Efficacy of testosterone-filled polydimethylsiloxane implants in maintaining plasma testosterone in rabbits. *J. Reprod. Fertil.*, **35,** 235.

VIVIANI, S., SANTORO, A., RAGUI, G., BONFANTE, V., BESTETTI, O. & BONADONNA, G. (1985). Gonadal toxicity after combination chemotherapy for Hodgkin's disease. Comparative results of MOPP vs. ABVD. *Eur. J. Cancer Clin. Oncol.*, **21,** 601.

WAXMAN, J. (1985). Cancer, chemotherapy, and fertility. *Br. Med. J.*, **290,** 1096.